# Supplementary material for: Decomposing the sources of SARS-CoV-2 fitness variation in the United States
Source: Virus Evol. 2021 Sep 2;7(2):veab073. doi: 10.1093/ve/veab073 (PMC8499931; doi:10.1093/ve/veab073)
Supplement: veab073_Supp [file veab073_supp.zip › SuppFig12_simulation_results.pdf]

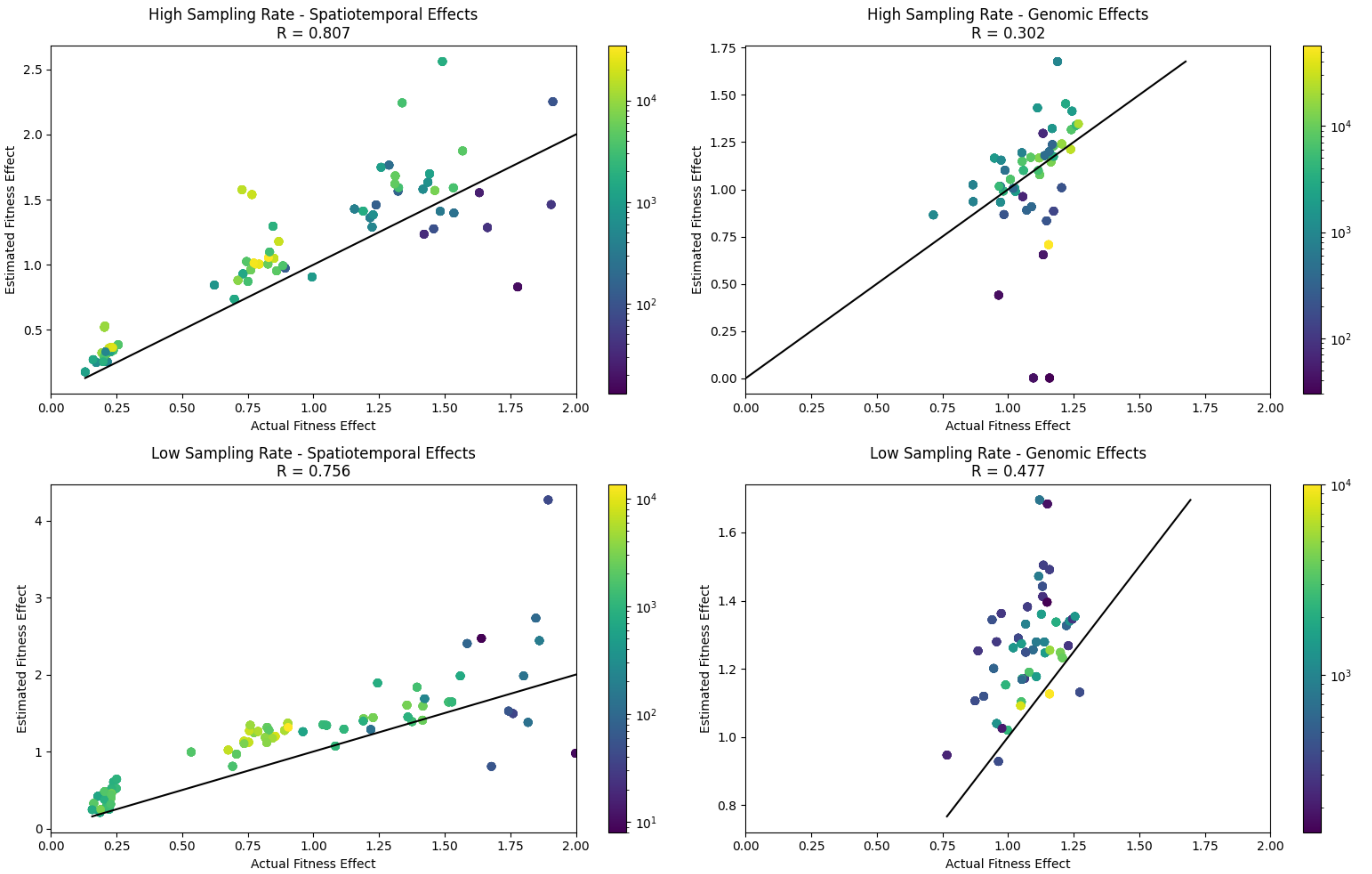

High Sampling Rate - Spatiotemporal Effects
R = 0.807
High Sampling Rate - Genomic Effects
R = 0.302
Low Sampling Rate - Spatiotemporal Effects
R = 0.756
Low Sampling Rate - Genomic Effects
R = 0.477
Estimated Fitness Effect
Actual Fitness Effect
