# Supplementary figures and images for: Decomposing the sources of SARS-CoV-2 fitness variation in the United States
Source: Virus Evol. 2021 Sep 2;7(2):veab073. doi: 10.1093/ve/veab073 (PMC8499931; doi:10.1093/ve/veab073)

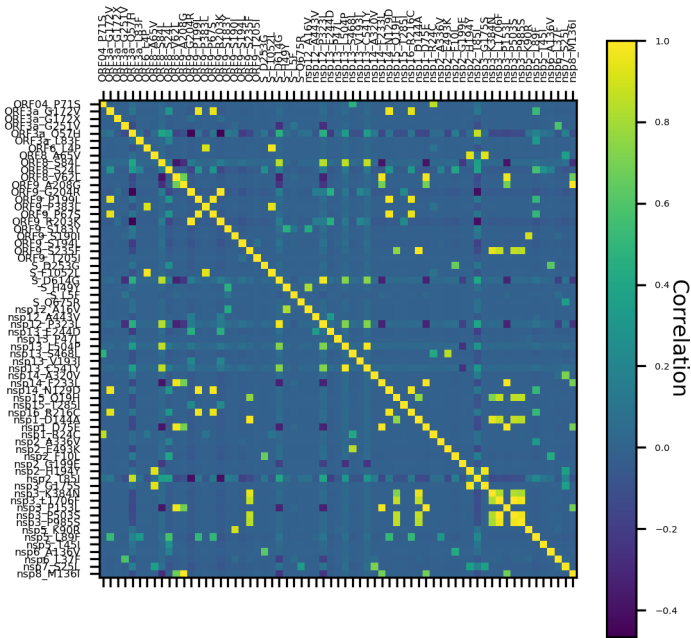

Supplement: veab073_Supp [file veab073_supp.zip › SuppFig1_variantCorrMatrix.pdf]

Estimated B.1.1.7 fitness

1.15

1.10

1.05

1.00

0.95

1X

5X

10X

Oversampling ratio

CA estimate

FL estimate

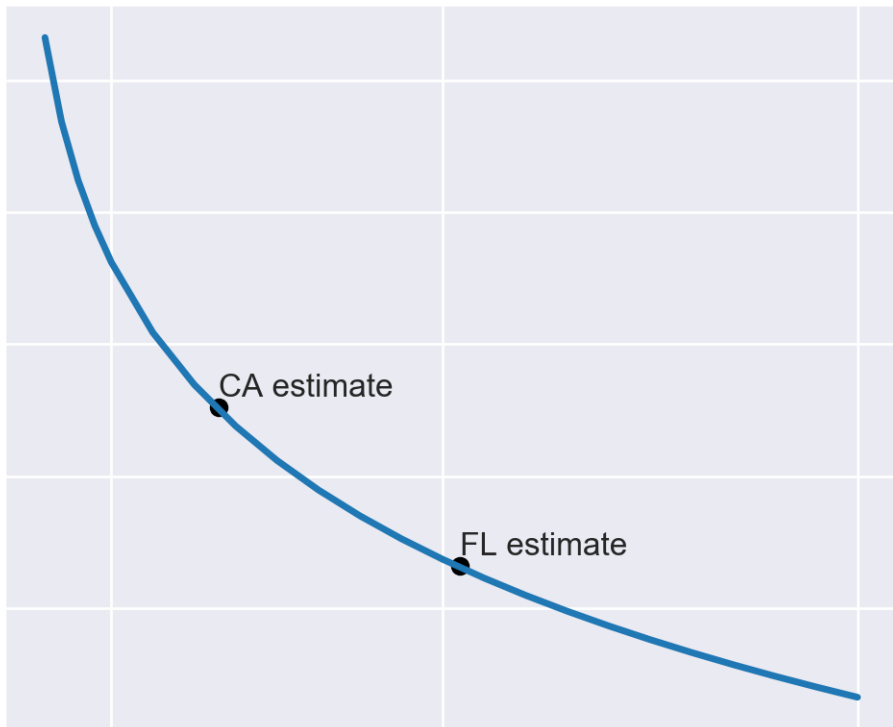

Supplement: veab073_Supp [file veab073_supp.zip › SuppFig10_B.1.1.7_sampleBiasSensitivityAnalysis.pdf]

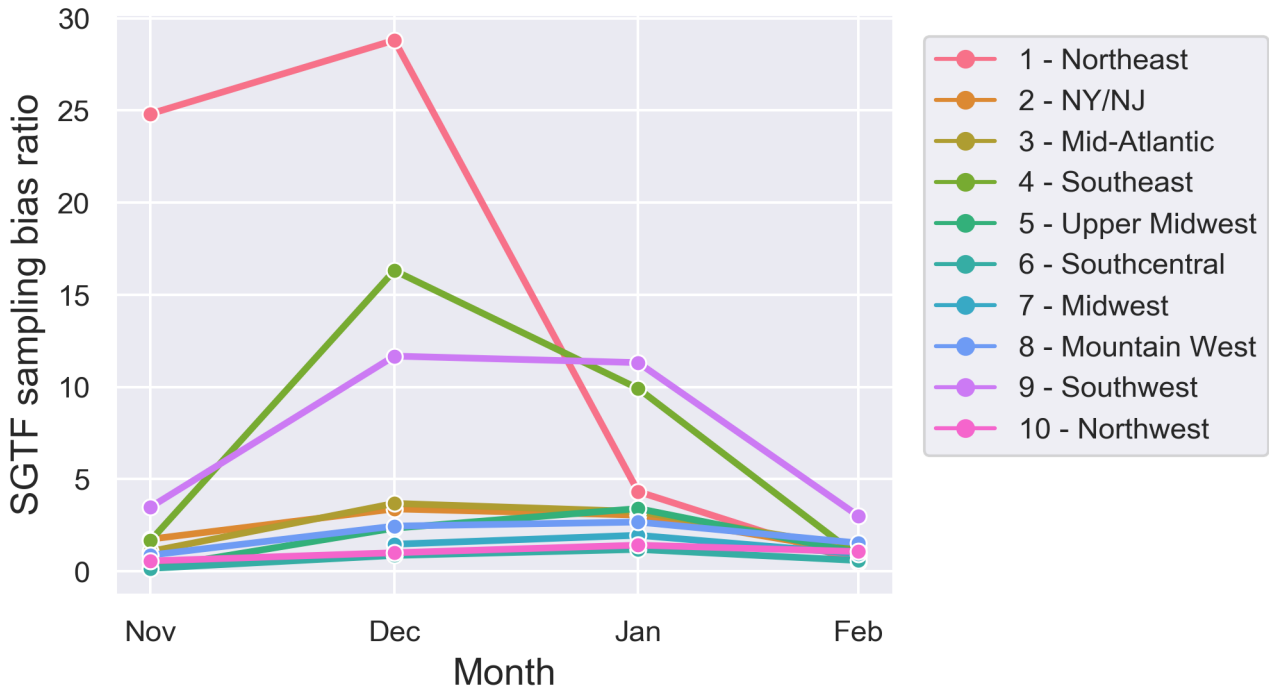

Supplement: veab073_Supp [file veab073_supp.zip › SuppFig11_spikeDeltaSamplingRatios.pdf]

Random branch effects  
 $R = 0.704$

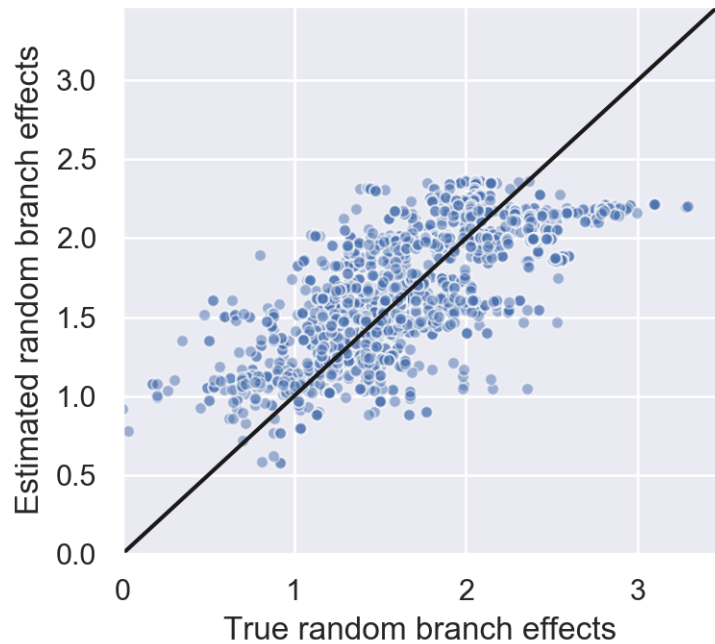

Random branch effects  
 $R = 0.658$

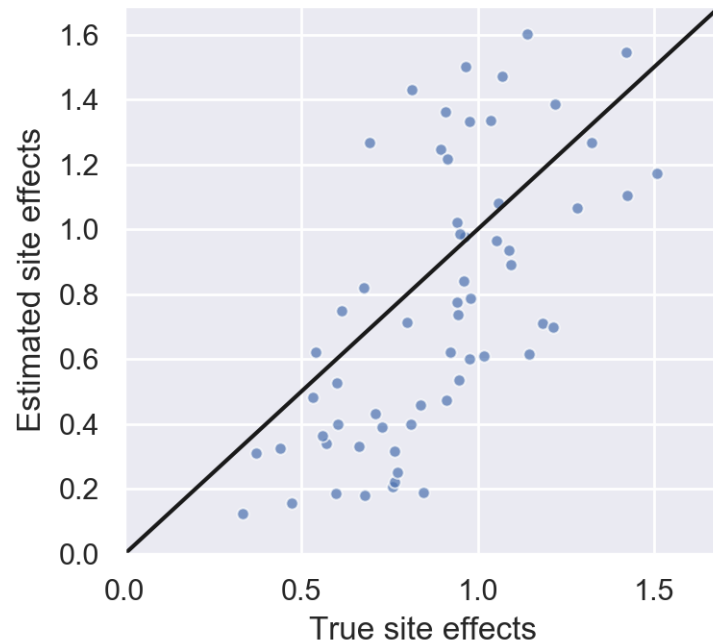

No random branch effects  
 $R = 0.485$

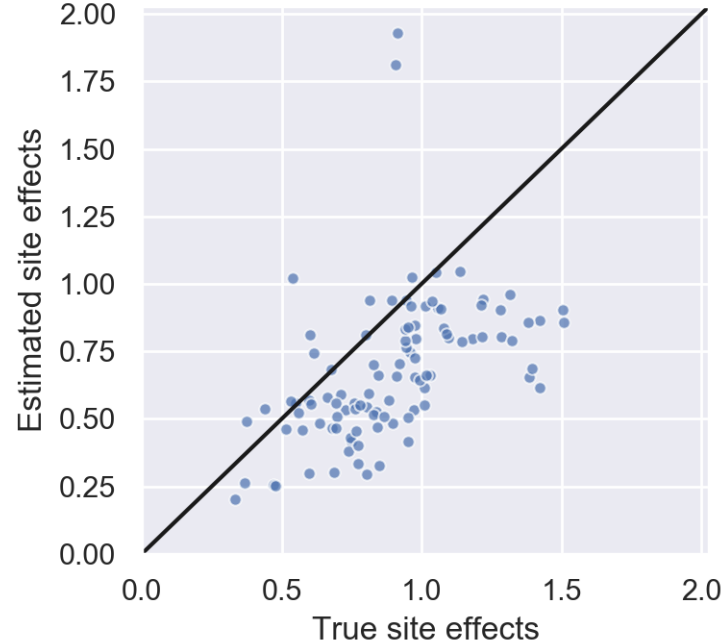

Supplement: veab073_Supp [file veab073_supp.zip › SuppFig13_randomBranchEffectsSims.pdf]

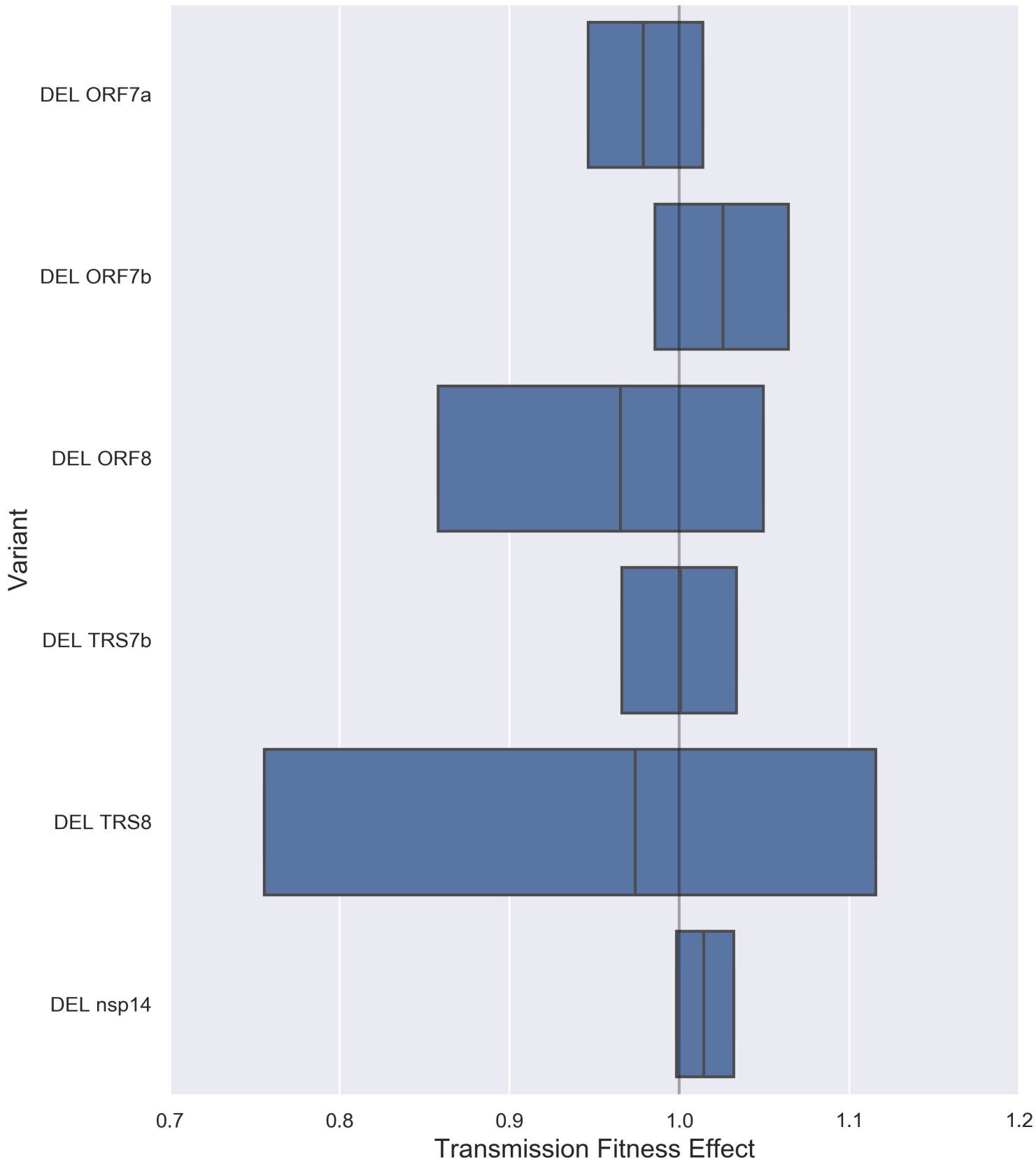

Supplement: veab073_Supp [file veab073_supp.zip › SuppFig2_structVariantsFit.pdf]

External intro ratio: 1.00

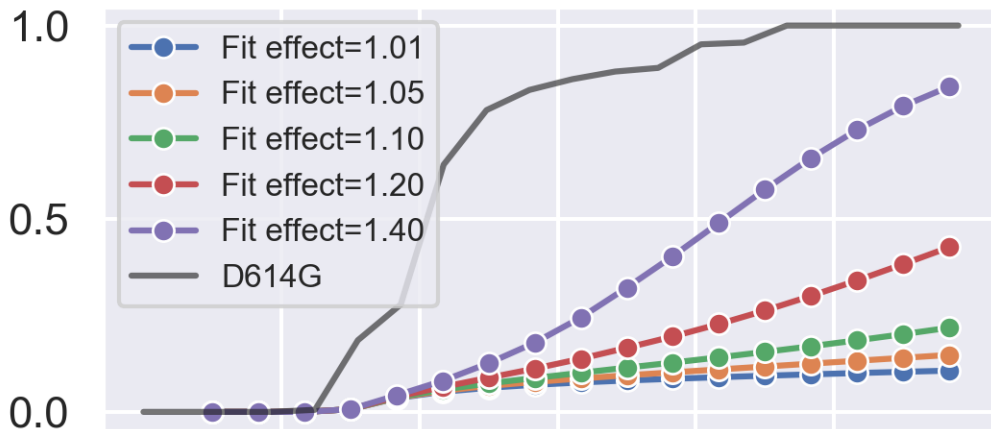

External intro ratio: 5.00

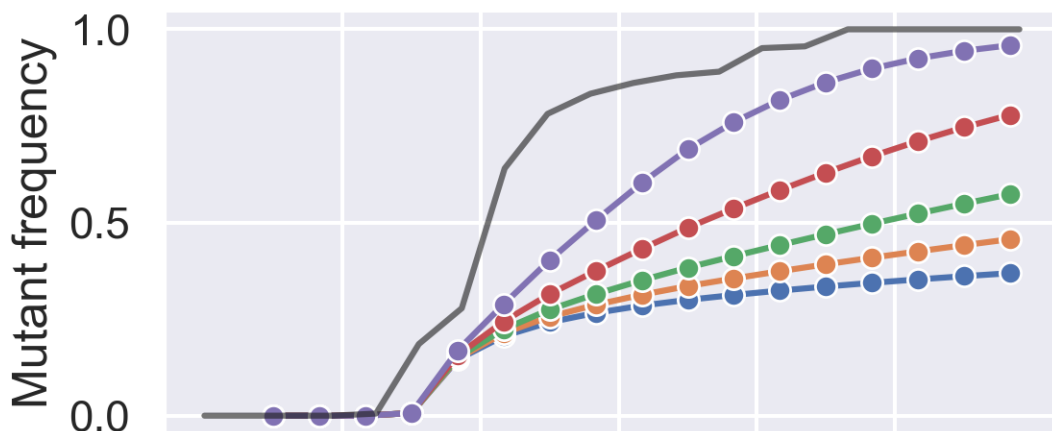

External intro ratio: 10.00

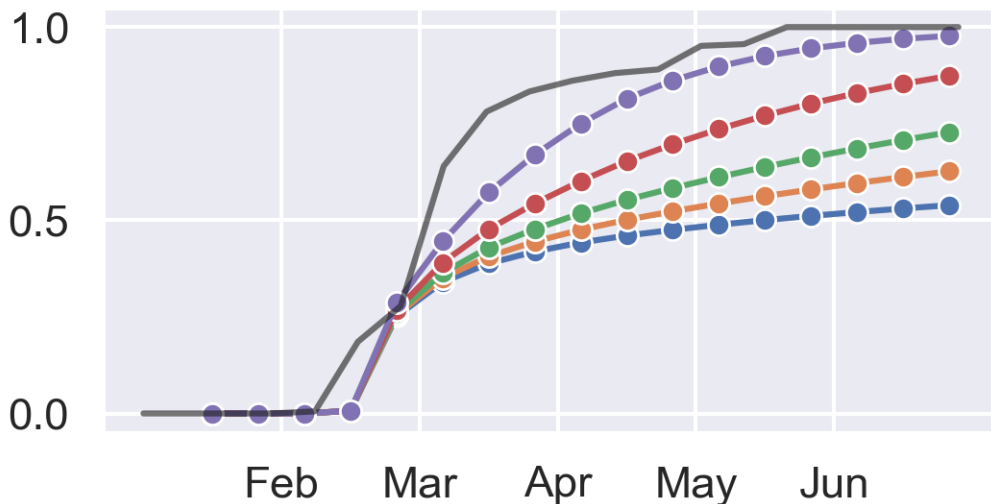

Supplement: veab073_Supp [file veab073_supp.zip › SuppFig3_SEIR_variant_traj.pdf]

$R = -0.009$

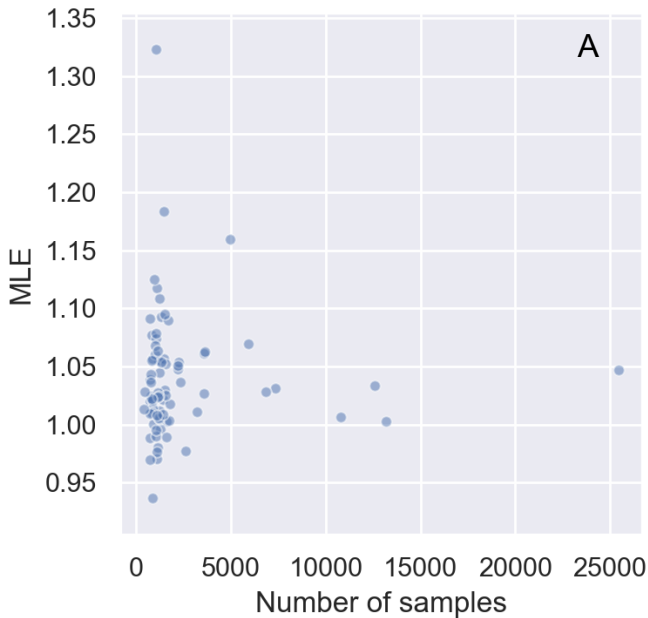

$R = -0.173$

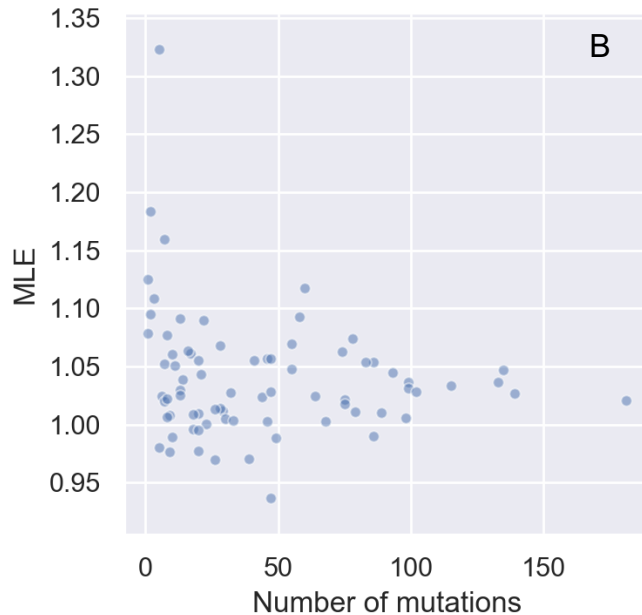

$R = -0.116$

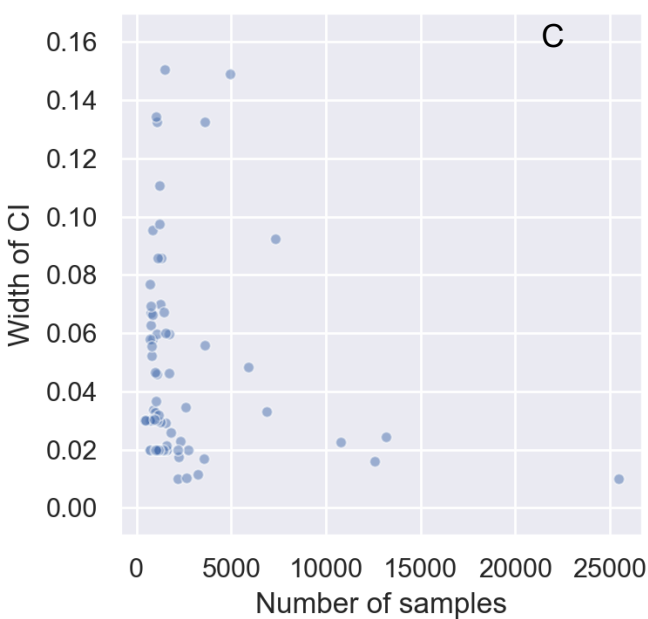

$R = -0.283$

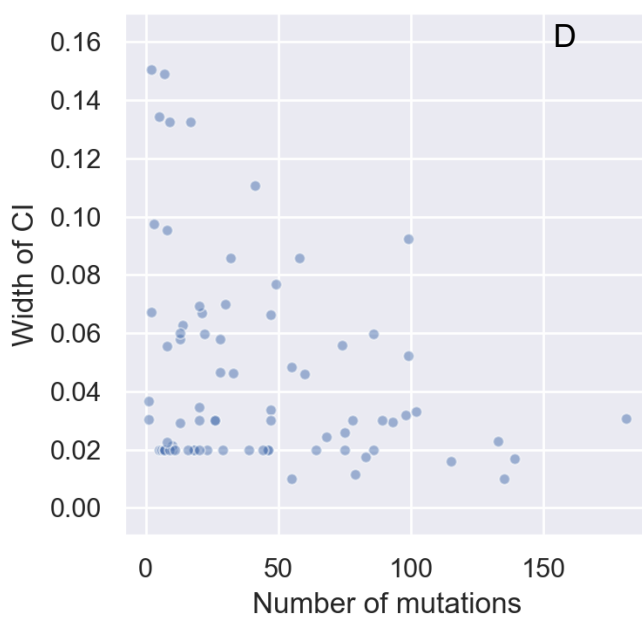

Supplement: veab073_Supp [file veab073_supp.zip › SuppFig4_aavFitnessEstVersusCounts.pdf]

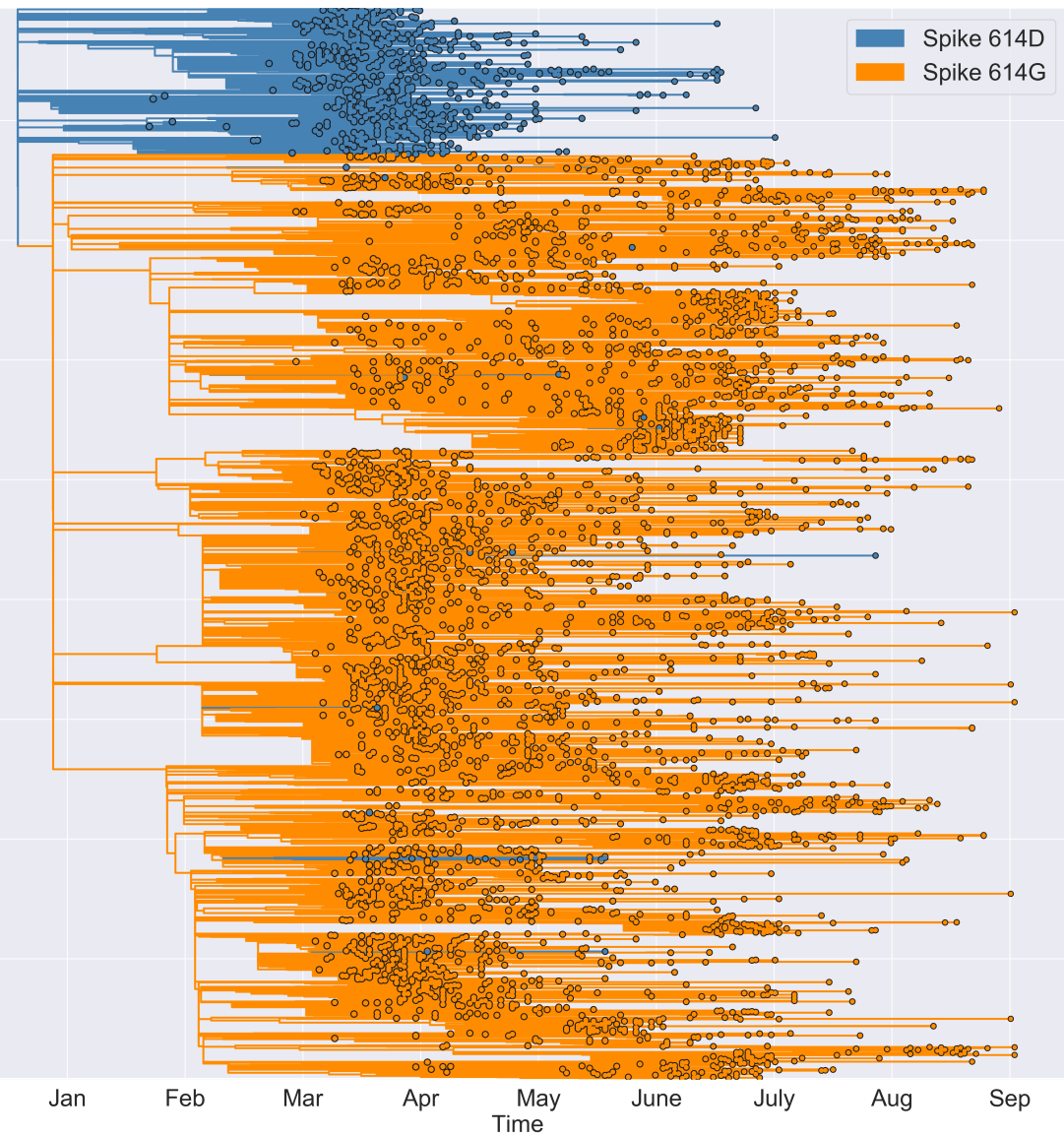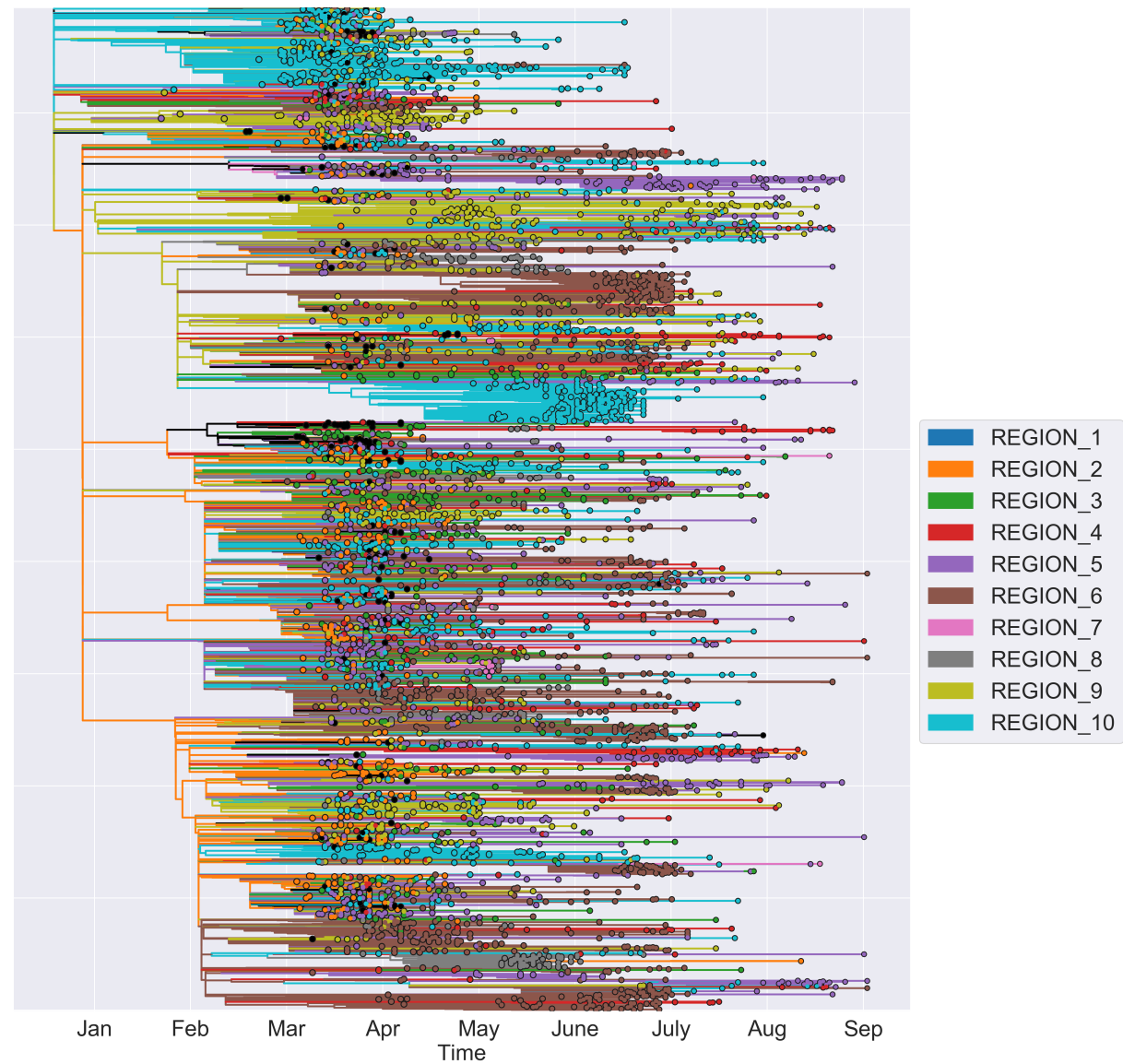

Supplement: veab073_Supp [file veab073_supp.zip › SuppFig5_ancS614RegionTree.pdf]

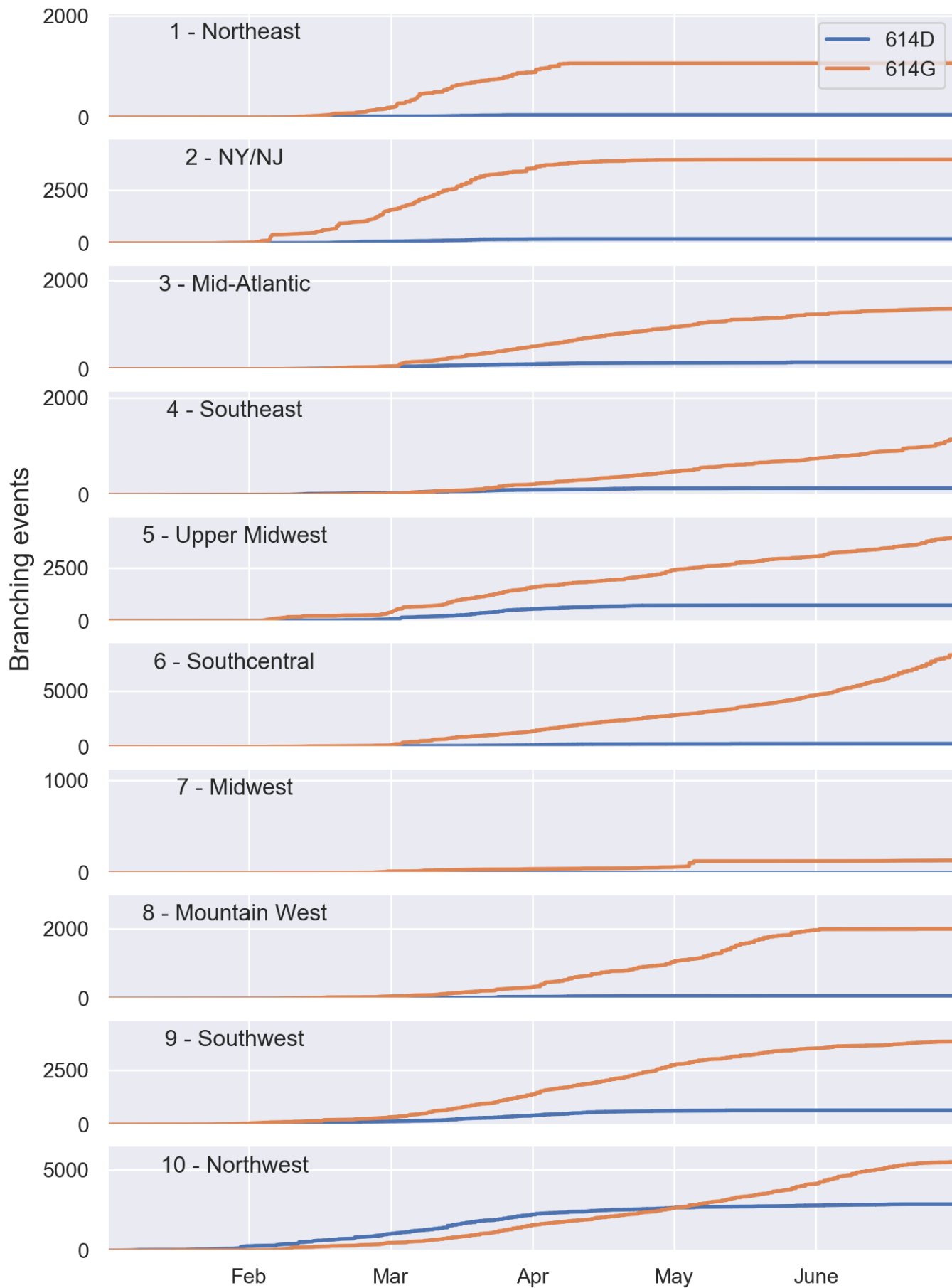

Supplement: veab073_Supp [file veab073_supp.zip › SuppFig6_cuml_transmission_byRegion.pdf]

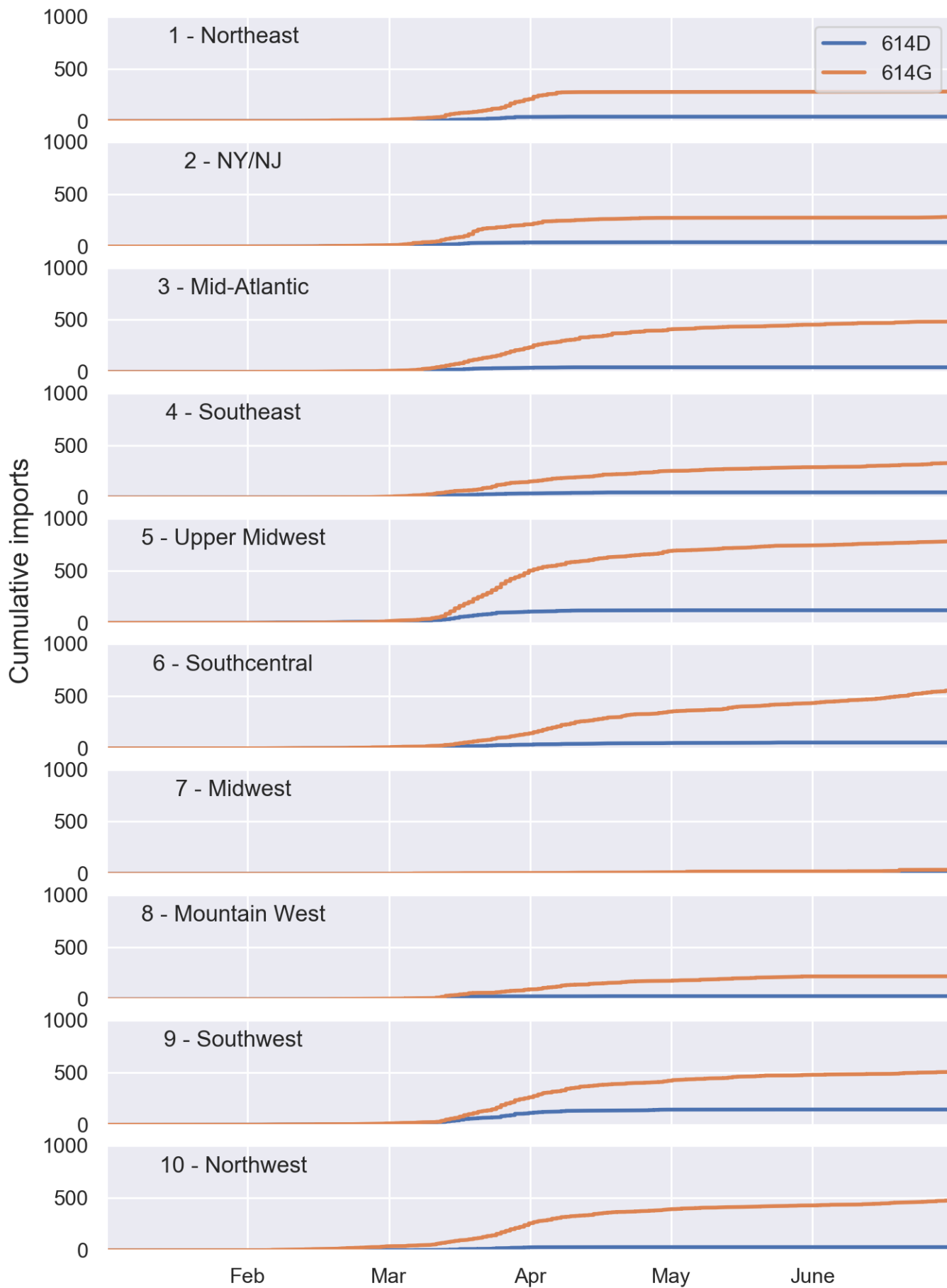

Supplement: veab073_Supp [file veab073_supp.zip › SuppFig7_cuml_imports_byRegion.pdf]

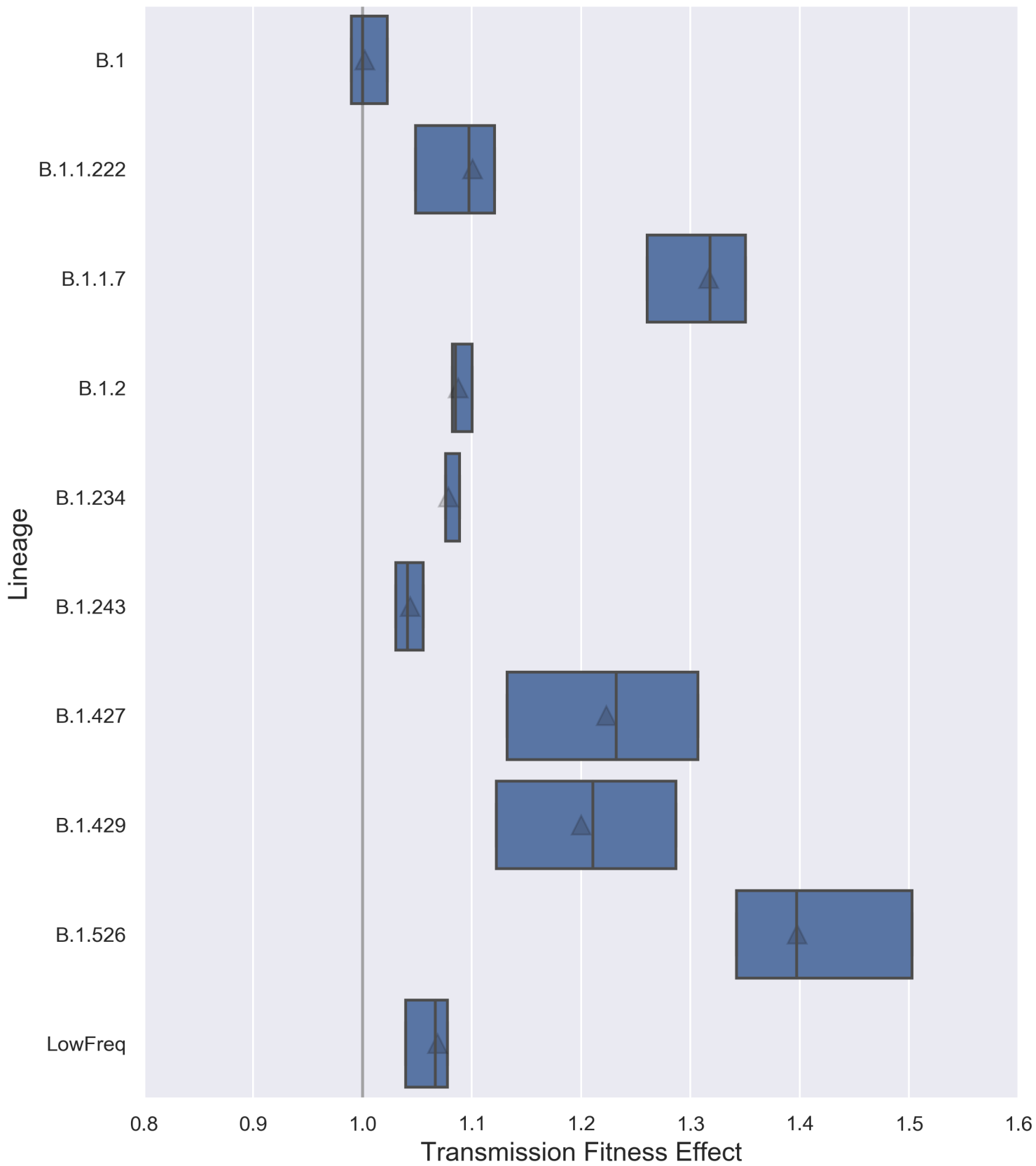

Supplement: veab073_Supp [file veab073_supp.zip › SuppFig8_pangoLineageFitEffects.pdf]

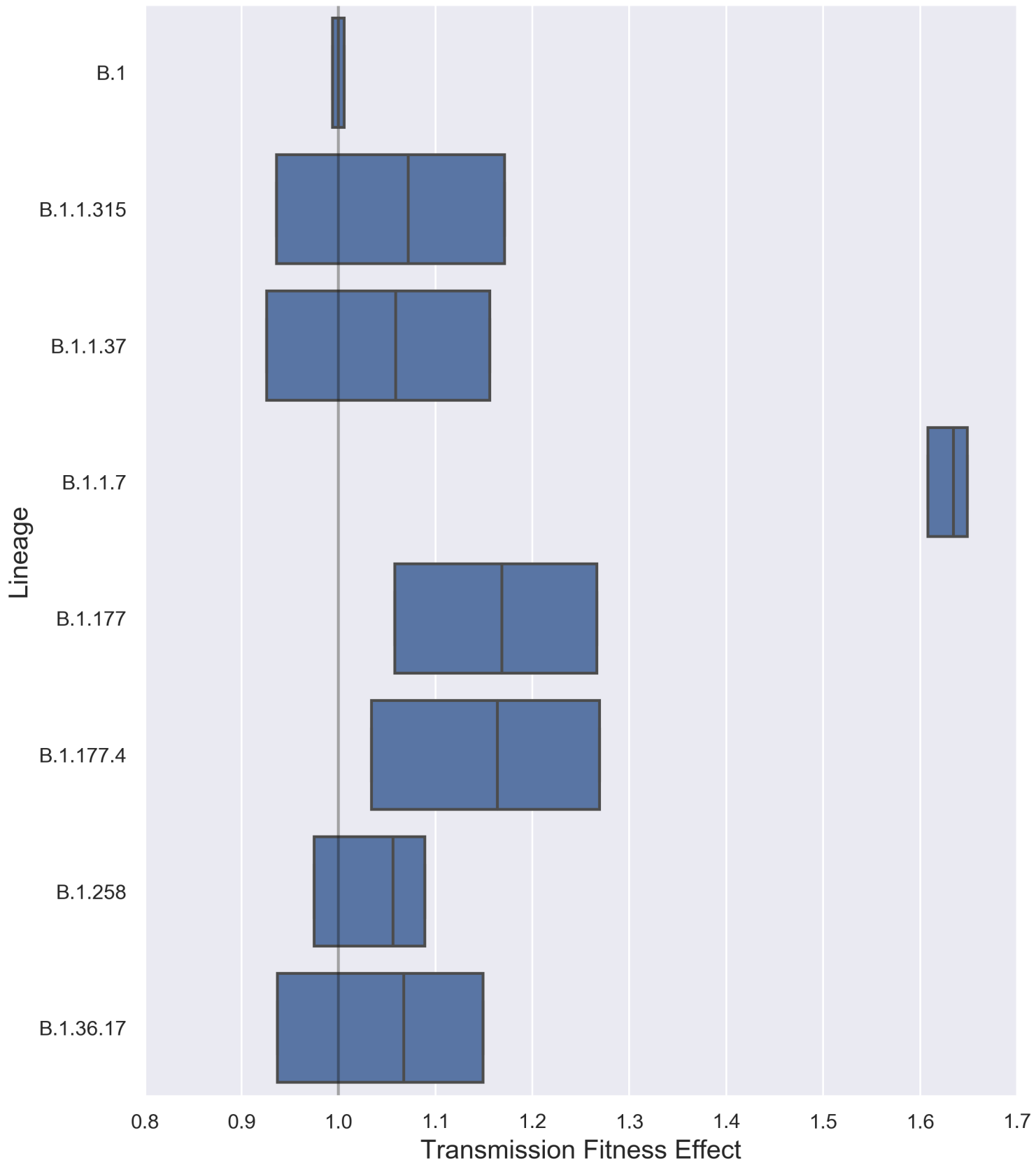

Supplement: veab073_Supp [file veab073_supp.zip › SuppFig9_UK_pangoLineageFitEffects.pdf]
